# Early and Long-Term Outcomes of Minimally Invasive Direct Coronary Artery Bypass in Elderly Patients: A Propensity Score-Matched Analysis

**DOI:** 10.3390/jcm15156043

**Published:** 2026-08-03

**Authors:** Lukman Amanov, Arian Arjomandi Rad, Sadeq Ali-Hasan-Al-Saegh, Thanos Athanasiou, Shivika Sharma, Jawad Salman, Ezin Deniz, Stefan Rümke, Bastian Schmack, Arjang Ruhparwar, Alina Zubarevich, Alexander Weymann

**Affiliations:** 1 Department of Cardiothoracic, Transplantation and Vascular Surgery, Hannover Medical School, Carl-Neuberg-Straße 1, 30625 Hannover, Germany; lukman.tm@gmail.com (L.A.); alsaegh.sadeq@gmail.com (S.A.-H.-A.-S.); ezin.deniz@mh-hannover.de (E.D.); ruemke.stefan@mh-hannover.de (S.R.); schmack.bastian@mh-hannover.de (B.S.); ruhparwar.arjang@mh-hannover.de (A.R.); weymann.alexander@googlemail.com (A.W.); 2 Department of Cardiothoracic Surgery, Oxford Heart Centre, John Radcliffe Hospital, Oxford University Hospitals NHS Foundation Trust, Oxford OX3 9DU, UK; 3 Department of Surgery and Cancer, Imperial College London, London SW7 2AZ, UK; t.athanasiou@imperial.ac.uk

**Keywords:** minimally invasive direct coronary artery bypass, MIDCAB, age, elderly, propensity score matching, left anterior descending artery, long-term survival, coronary revascularization

## Abstract

**Background:** Advancing age is incorporated as a strong risk variable in EuroSCORE II and is consistently associated with adverse outcomes after conventional coronary artery bypass grafting (CABG). Whether minimally invasive direct coronary artery bypass (MIDCAB)—which avoids both sternotomy and cardiopulmonary bypass—modifies this age-related risk in patients with single-vessel or LAD-predominant coronary artery disease remains insufficiently characterised. **Methods:** We retrospectively analysed 350 consecutive patients who underwent MIDCAB at Hannover Medical School between July 1999 and April 2025 (follow-up to April 2025). Elderly patients (age ≥70 years; n = 117) were compared with younger patients (age <70 years; n = 233) before and after 1:1 propensity score matching using greedy nearest-neighbour matching with a caliper of 0.2 × SD of the logit propensity score; age was excluded from the propensity model as it represented the exposure variable. A pre-specified sensitivity propensity model that additionally excluded EuroSCORE II (because EuroSCORE II contains an age component) was also evaluated. The primary endpoint was all-cause long-term mortality; secondary endpoints included perioperative complications and in-hospital outcomes. Long-term survival was assessed by Kaplan–Meier analysis and multivariable Cox proportional hazards regression, with a pre-specified parsimonious Cox model (age, LVEF, renal impairment) and cluster-robust standard errors on matched-pair identifiers. **Results:** Matching produced 109 pairs with excellent covariate balance (all standardized mean differences < 0.20). MIDCAB was completed without intraoperative conversion in all patients. No 30-day mortality, perioperative stroke, or new postoperative dialysis was observed in either age stratum (Clopper–Pearson 95% CI 0.00–3.33% for each zero-event outcome). Perioperative complications—including new-onset atrial fibrillation, re-exploration for bleeding, and intensive care unit and hospital length of stay—did not differ significantly between elderly and younger patients in the matched cohort (Newcombe 95% CI for risk differences all crossing zero; McNemar’s exact tests non-significant for all matched-pair binary endpoints; Hodges–Lehmann median difference for hospital length of stay +1.0 day, bootstrap 95% CI 0.0–1.0 days). At a median follow-up of 19.0 years (IQR 11.5–24.3; reverse Kaplan–Meier potential median 19.7 years), all-cause mortality was higher in the elderly (20.2% vs. 5.5%, *p* = 0.002; log-rank *p* = 0.001; exact McNemar’s *p* = 0.0025 for the matched-pair mortality endpoint). After multivariable adjustment, elderly age (≥70 years) was independently associated with long-term mortality (adjusted hazard ratio 4.48, 95% CI 1.79–11.20, *p* = 0.001), as was EuroSCORE II (HR 2.40 per unit, *p* = 0.034); a pre-specified parsimonious model (age, LVEF, renal impairment) with pair-cluster robust standard errors yielded an essentially identical adjusted HR for elderly age of 4.08 (95% CI 1.65–10.04, *p* = 0.002), and a sensitivity propensity model without EuroSCORE II yielded HR 3.95 (95% CI 1.68–9.29, *p* = 0.002). **Conclusions:** In this propensity-matched analysis with a median follow-up of ~19 years, MIDCAB was associated with excellent observed perioperative outcomes in appropriately selected elderly patients (no 30-day mortality, stroke, or new dialysis observed; upper 95% CI 3.3%) and no evidence of an excess of major in-hospital complications compared with younger patients within the statistical resolution of the cohort. The long-term mortality excess in the elderly is consistent with age-related life-expectancy curves in the source population; cause-specific mortality was not available in this cohort. External benchmarks from large MIDCAB cohorts in which long-term survival approximates that of the age-matched general population support this interpretation indirectly. These findings support MIDCAB as a feasible revascularization strategy associated with favourable observed early outcomes and long-term survival consistent with published MIDCAB literature, in appropriately selected elderly patients with single-vessel or LAD-predominant coronary artery disease treated at experienced centres.

## 1. Introduction

The global population is ageing rapidly, and an increasing proportion of patients referred for coronary artery bypass grafting (CABG) are over 70 years of age [1,2]. Coronary artery bypass grafting has been the cornerstone of surgical revascularization for more than five decades, and its survival benefit—particularly when the left internal mammary artery (LIMA) is anastomosed to the left anterior descending (LAD) coronary artery—remains unmatched by any alternative therapy for selected anatomical subsets [3,4]. However, conventional CABG via median sternotomy with cardiopulmonary bypass is associated with substantial perioperative trauma—including risks of sternal wound complications, neurocognitive injury, transfusion requirements, and prolonged recovery—that are amplified in older patients with reduced physiological reserve.

Advancing age is one of the most consistent predictors of adverse outcome after conventional CABG [5,6]. Large registry analyses have demonstrated higher in-hospital and 30-day mortality, increased rates of stroke and renal dysfunction, longer ICU and hospital stay, and slower functional recovery in elderly patients undergoing sternotomy CABG [7,8]. Age is incorporated as a strong continuous risk variable in both the Society of Thoracic Surgeons score and EuroSCORE II [1]. The GOPCABE randomised trial, which compared off-pump and on-pump CABG in patients aged ≥75 years, demonstrated equivalent outcomes between the two approaches but confirmed that the overall morbidity of conventional cardiac surgery remains substantial in this age group [9]. Importantly, a secondary analysis of GOPCABE showed that, once baseline risk is accounted for, female sex is no longer an independent risk factor in elderly patients, suggesting that age itself—together with the cumulative burden of comorbidity—drives much of the observed perioperative risk in older surgical candidates [10].

Minimally invasive direct coronary artery bypass (MIDCAB), introduced by Calafiore and colleagues in 1996 [11], addresses isolated LAD disease—or the LAD component of multivessel disease in the context of hybrid revascularization—through a small left anterolateral thoracotomy and off-pump grafting of the LIMA to the LAD. By avoiding both sternotomy and cardiopulmonary bypass, MIDCAB substantially reduces blood transfusion requirements, ventilation time, length of stay, and sternal wound complications, while preserving the long-term durability of the LIMA-LAD graft, with reported patency rates exceeding 90% at 10–15 years [3,4,5]. These attributes might be expected to disproportionately benefit subgroups in whom conventional CABG carries the highest relative risk—most notably, elderly patients.

Data on age-specific outcomes after MIDCAB remain limited. The largest single-centre experience, reported by Davierwala and colleagues over a 20-year period at the Leipzig Heart Center, demonstrated an overall in-hospital mortality of 0.9% across 2667 patients, with stable outcomes despite a progressive worsening of the demographic risk profile, including increasing patient age, over time [5]. Manuel and colleagues recently demonstrated that MIDCAB patients with isolated LAD disease return to an age- and sex-matched standardised mortality ratio of 0.94—that is, their survival approximates that of the general population [6]. However, propensity-matched analyses specifically comparing elderly and younger MIDCAB patients with long follow-up are scarce. Given the growing referral of elderly patients for surgical coronary revascularization, robust age-stratified analyses are needed to inform clinical decision-making and risk communication.

The present study was designed to evaluate age-specific outcomes after MIDCAB performed for single-vessel or LAD-predominant coronary artery disease in a single-centre cohort with a median follow-up approaching two decades. To minimise confounding by baseline comorbidity differences between age groups, we applied 1:1 propensity score matching—excluding age from the propensity model, since age was the exposure variable—and assessed perioperative complications, in-hospital outcomes, and long-term all-cause mortality. We further used multivariable Cox regression to determine whether age ≥70 years is an independent predictor of long-term mortality after MIDCAB.

## 2. Materials and Methods

### 2.1. Study Design and Patient Population

This was a retrospective, single-centre, observational cohort study of consecutive patients who underwent MIDCAB at Hannover Medical School between July 1999 and April 2025, with follow-up extending to April 2025. Eligible patients were adults (≥18 years) in whom MIDCAB was performed for single-vessel coronary artery disease (stenosis of the LAD) or as the surgical component of a hybrid revascularization strategy for multivessel disease with an LAD lesion. The decision to perform MIDCAB was individualised on the basis of coronary anatomy, comorbidity profile, multidisciplinary heart team consensus, and patient preference, in accordance with the 2018 ESC/EACTS guidelines on myocardial revascularization [12].

The selection process warrants explicit description. MIDCAB was offered on a case-by-case basis by the multidisciplinary Heart Team after coronary angiographic assessment. The primary indication was isolated proximal LAD disease unsuitable for percutaneous coronary intervention or LAD-predominant multivessel disease considered for hybrid revascularization. Anatomical prerequisites—an adequately sized, epicardial LAD; absence of prior left thoracotomy or significant left pleural adhesions; and acceptable respiratory reserve for single-lung ventilation—had to be satisfied. Patients with prohibitive frailty, unresected primary lung neoplasia in the left hemithorax, or an anatomically hostile chest wall (severe pectus deformity, extensive post-radiotherapy fibrosis) were not offered the procedure. Importantly, elderly patients in whom the Heart Team judged conventional sternotomy CABG to be prohibitively high risk were preferentially offered MIDCAB or percutaneous intervention, which by design enriches the elderly MIDCAB cohort for a comparatively favourable non-cardiac risk profile. This selection bias is made explicit in Section 4.6 (Limitations). A patient flow diagram is provided in Supplementary Figure S1.

Patients were stratified by chronological age at the time of surgery: elderly (≥70 years; n = 117) versus younger (<70 years; n = 233). The age cutoff of 70 years was selected a priori because it represents the conventional threshold for ‘elderly’ in cardiac surgery risk-stratification literature, balances clinical relevance with statistical power, and yields adequate sample sizes for propensity-matched comparison [13,14]. These study was conducted in accordance with the Declaration of Helsinki. Ethical review and approval were waived for this retrospective, non-interventional study because it used fully anonymized patient data. In accordance with German regulations, including §15 of the professional code of conduct of the German Medical Association and the corresponding regulations of the State Medical Chambers, retrospective studies based exclusively on anonymized data do not require prior ethics committee approval.

### 2.2. Surgical Technique

All MIDCAB procedures were performed under general anaesthesia with single-lumen endotracheal intubation, in the technique originally described by Calafiore and colleagues [11]. Patients were positioned supine with slight elevation of the left hemithorax. A small (approximately 5–8 cm) left anterolateral thoracotomy was performed in the fourth or fifth intercostal space, tailored to coronary anatomy and operator preference. The LIMA was harvested under direct vision using a dedicated soft-tissue retractor, without rib resection. After systemic heparinisation, the LAD was exposed and stabilised with an epicardial stabiliser. The LIMA-to-LAD anastomosis was constructed off-pump on the beating heart using running monofilament suture. Intraoperative graft patency was verified by direct inspection and by transit-time flow measurement, which was performed selectively rather than systematically. The criterion for use was any anastomosis in which palpation or direct inspection did not provide unequivocal reassurance of graft patency. No intraoperative conversions to conventional sternotomy or cardiopulmonary bypass occurred in the present cohort.

### 2.3. Data Collection and Definitions

Demographic, clinical, operative, and outcome data were extracted from the institutional electronic medical record and prospectively maintained surgical database. Preoperative variables included age, sex, EuroSCORE II [1], left ventricular ejection fraction (LVEF), urgency of operation (elective, urgent or emergency), coronary artery disease pattern (single-, two-, or three-vessel involvement), recent myocardial infarction (<90 days), ST-elevation and non-ST-elevation myocardial infarction, prior percutaneous coronary intervention (PCI), smoking status, diabetes mellitus (non-insulin and insulin dependent), arterial hypertension, hyperlipidemia, renal impairment, and preoperative dialysis.

Key clinical variable definitions were as follows. Renal impairment was defined as estimated glomerular filtration rate < 60 mL/min/1.73 m^2^ by the CKD-EPI equation (KDIGO CKD stage ≥ G3a) or dependence on chronic dialysis at the time of surgery. Diabetes mellitus was defined as documented physician diagnosis with active hypoglycaemic therapy. New-onset atrial fibrillation (NOAF) was defined as any atrial fibrillation detected on continuous ECG monitoring during hospitalisation in a patient without a prior documented history of atrial fibrillation. Perioperative stroke was defined as any new focal neurological deficit lasting >24 h with a corresponding imaging correlate. Postoperative myocardial infarction was defined per the Fourth Universal Definition (type 5 in the perioperative setting). Re-exploration for bleeding was defined as return to the operating theatre for surgical haemostasis within 48 h of the index procedure. Renal replacement therapy was defined as any form of dialysis initiated de novo postoperatively.

Intraoperative variables included operative time, intraoperative red blood cell (RBC) and platelet transfusion, and conversion to conventional CABG. Postoperative in-hospital endpoints included length of intensive care unit (ICU) stay, duration of mechanical ventilation, hospital length of stay, new-onset atrial fibrillation (NOAF), re-exploration for bleeding, new postoperative dialysis, stroke, postoperative myocardial infarction, peak creatine kinase (CK), CK-MB and troponin, postoperative RBC transfusion in the ICU, and the need for postoperative coronary angiography. The primary endpoint was all-cause long-term mortality. Secondary long-term endpoints were 30-day mortality (death within 30 days of surgery), long-term myocardial infarction, and the patency of the LIMA-LAD anastomosis among patients who underwent follow-up coronary angiography.

### 2.4. Follow-Up

Follow-up was obtained from outpatient clinic visits, written correspondence with referring cardiologists and general practitioners, and direct telephone contact with patients or relatives where necessary. Mortality status was cross-checked against national registry data. Follow-up duration was calculated from the date of surgery to the date of death or last contact. Follow-up completeness was 100% for the primary endpoint of all-cause mortality; the potential median follow-up estimated by the reverse Kaplan–Meier method was 19.7 years overall (20.7 years in the matched elderly group; 19.9 years in the matched younger group), confirming that the observed median follow-up of 19.0 years reflects administrative censoring at the study end rather than premature loss to follow-up.

### 2.5. Statistical Analysis

Continuous variables were assessed for normality by visual inspection and Shapiro–Wilk testing and are reported as mean ± standard deviation (SD) or median with interquartile range (IQR), as appropriate. Categorical variables are reported as counts and percentages. Between-group comparisons used Welch’s *t*-test or the Mann–Whitney U test for continuous variables, and the chi-square (χ^2^) or Fisher’s exact test for categorical variables, with the latter applied when expected cell counts were below five.

For endpoints in the matched cohort, analyses appropriate to the paired design were additionally performed: McNemar’s exact test for binary matched-pair outcomes; Hodges–Lehmann estimates of median between-group differences with bootstrap 95% confidence intervals (2000 resamples) for continuous outcomes; exact (Clopper–Pearson) 95% confidence intervals for individual event rates; and Newcombe hybrid-score 95% confidence intervals for between-group risk differences of rare events, including those with zero counts.

To minimise confounding by baseline differences between age groups, a propensity score for elderly age (≥70 years) was estimated using multivariable logistic regression. Critically, age itself was not included as a covariate in the propensity model, as it represented the exposure variable; including the exposure in the propensity model would have produced perfect separation and precluded matching. Variables entered into the propensity model were selected a priori as clinically relevant determinants of perioperative risk and long-term outcome other than age, and included female sex, EuroSCORE II, LVEF, single-, two-, and three-vessel coronary artery disease, recent myocardial infarction, prior PCI, smoking, diabetes mellitus, arterial hypertension, hyperlipidemia, renal impairment, and non-elective status. Predictors were standardised prior to model fitting. One-to-one matching without replacement was then performed using a greedy nearest-neighbour algorithm on the logit of the propensity score, with a caliper width of 0.2 × the standard deviation of the logit propensity score, as recommended by Austin [2]. Balance was assessed before and after matching using absolute standardized mean differences (|SMD|), with |SMD| < 0.20—and ideally < 0.10—considered indicative of adequate balance [15].

Because EuroSCORE II incorporates age as one of its components and could therefore reintroduce a small amount of age information into the propensity model, a pre-specified sensitivity propensity score analysis was performed with EuroSCORE II excluded from the model, retaining all other covariates. Then, 1:1 matching was performed with the same caliper. The sensitivity analysis is reported alongside the primary analysis.

Long-term survival was estimated using the Kaplan–Meier method and compared between age groups using the log-rank test, both in the unmatched and matched cohorts. Kaplan–Meier survival estimates with 95% confidence intervals were reported at 1, 5, 10, 15, and 20 years. Independent predictors of long-term all-cause mortality were assessed by multivariable Cox proportional hazards regression, including elderly age (≥70 years), female sex, EuroSCORE II, LVEF, diabetes mellitus, renal impairment, and a history of preoperative myocardial infarction. In view of the modest number of events (28 deaths in the matched cohort) and to avoid overfitting, a pre-specified parsimonious Cox model restricted to three biologically motivated covariates—age ≥70 years (the exposure), LVEF, and renal impairment—was additionally fitted (events per variable ~9). To correctly reflect the matched-pair design, robust standard errors clustered on the matched-pair identifier were applied. A univariate matched-pair Cox model (age ≥70 only with pair-cluster robust standard errors) was also fitted. The proportional hazards assumption was examined by inspection of Schoenfeld residuals. Results are expressed as hazard ratios (HRs) with 95% confidence intervals (CIs). A sensitivity Cox analysis was repeated in the full unmatched cohort to assess robustness.

All statistical tests were two-sided and a *p*-value < 0.05 was considered statistically significant. Analyses were performed in Python 3 using the pandas, NumPy, scikit-learn, SciPy, statsmodels, and lifelines libraries.

## 3. Results

### 3.1. Study Population and Baseline Characteristics

Between July 1999 and April 2025, 350 consecutive patients underwent MIDCAB at Hannover Medical School and were included in the analysis; follow-up extended to April 2025. No patients were excluded (see Supplementary Figure S1). Of these, 117 (33.4%) were ≥70 years of age (elderly) and 233 (66.6%) were <70 years of age (younger). The MIDCAB procedure was completed in all 350 patients without intraoperative conversion to conventional CABG. Single-vessel coronary artery disease involving the LAD was present in 227 patients (64.9%), whereas multivessel disease (in which MIDCAB was performed to address the LAD lesion) was present in 123 patients (35.1%). In multivessel-disease patients, MIDCAB was performed to address the LAD lesion; the completeness of revascularization of the non-LAD territory (whether by staged percutaneous coronary intervention as part of a hybrid strategy or by medical management of residual disease) was not systematically captured in the database and is acknowledged as a limitation (Section 4.6). The median follow-up was 19.0 years (IQR 11.8–23.9), providing a long-term perspective on survival and graft-related outcomes; the reverse Kaplan–Meier potential median follow-up was 19.7 years (see Supplementary Figure S2), confirming administrative rather than premature censoring.

By design, elderly patients were substantially older than younger patients (mean 75.4 ± 4.1 vs. 57.6 ± 8.3 years, *p* < 0.001). Before matching, elderly patients had a modestly higher EuroSCORE II (median 0.9 [IQR 0.8–1.2] vs. 0.8 [0.7–1.1], *p* < 0.001), a slightly lower LVEF (56.4 ± 5.3% vs. 57.9 ± 5.6%, *p* = 0.018), and were more likely to be female (36.8% vs. 25.3%, *p* = 0.036). Other baseline characteristics—including the distribution of single- versus multivessel disease, prior PCI, recent myocardial infarction, diabetes, arterial hypertension, hyperlipidemia, smoking, and renal impairment—did not differ significantly between age groups, with all SMDs < 0.30 (Table 1). The cohort assembly and matching process is summarised in the patient flow diagram (Supplementary Figure S1).

### 3.2. Propensity Score Matching

**Figure 1:**
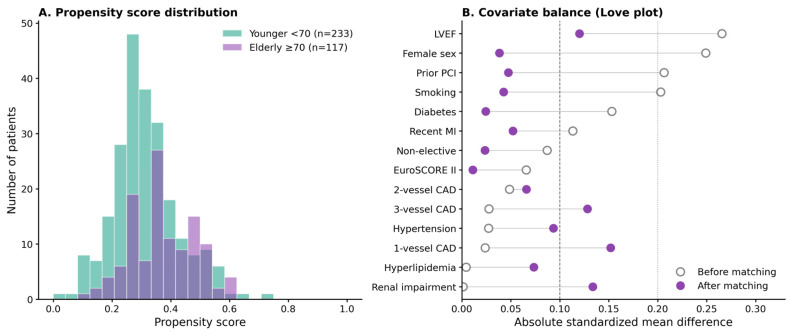
Propensity score distribution and covariate balance. (**A**) Histogram of estimated propensity scores in elderly (purple) and younger (teal) patients before matching, demonstrating substantial overlap. (**B**) Love plot of absolute standardized mean differences for all matching covariates before (open circles) and after (purple circles) 1:1 propensity score matching. Dashed line: |SMD| = 0.10; dotted line: |SMD| = 0.20. Age was excluded from the propensity model as it represented the exposure variable.

Greedy nearest-neighbour matching without replacement was performed using a caliper of 0.2× the SD of the logit propensity score (=0.111), as recommended by Austin [2]. Age itself was excluded from the propensity model since it represented the exposure variable. Of the 117 elderly patients, 109 (93.2%) were successfully matched to a younger counterpart, producing a final matched cohort of 218 patients (109 per group). The propensity score distributions in the two groups overlapped substantially before matching (Figure 1A). After matching, all absolute standardized mean differences fell well below the conventional 0.20 threshold, with most below 0.15, indicating excellent covariate balance [15] (Figure 1B). Post-matching, elderly and younger patients were comparable in EuroSCORE II (median 0.9 [0.7–1.2] vs. 0.9 [0.7–1.1], *p* = 0.136), LVEF (56.7 ± 5.3% vs. 57.3 ± 5.5%, *p* = 0.376), female sex (34.9% vs. 36.7%, *p* = 0.888), and across all comorbid conditions and urgency categories (Table 2). The matched cohort retained the large by-design age difference (75.4 ± 4.1 vs. 58.2 ± 8.2 years, *p* < 0.001).

As a pre-specified sensitivity analysis, an alternative propensity model excluding EuroSCORE II (which itself contains age as one of its components) was fitted using the remaining 13 covariates. This alternative model produced 108 matched pairs (caliper 0.108), with comparable balance across all covariates, and yielded log-rank *p* = 0.001 for long-term all-cause mortality; the corresponding parsimonious Cox HR for elderly age was 3.95 (95% CI 1.68–9.29, *p* = 0.002), essentially identical to the primary analysis (see Section 3.6).

### 3.3. Intraoperative Outcomes

In the propensity-matched cohort, operative time was numerically longer in elderly patients but did not differ significantly between groups (130.1 ± 35.1 vs. 122.5 ± 30.9 min, *p* = 0.091). MIDCAB was performed as an off-pump procedure in all cases, with a LIMA-to-LAD anastomosis. No intraoperative conversion to conventional CABG occurred in either group. Intraoperative RBC transfusion requirements were comparable: 20.2% of elderly and 29.4% of younger patients received at least one unit (*p* = 0.158), with similar median transfusion volumes (both 0 units; *p* = 0.102). Platelet transfusion was rarely required and did not differ between groups (*p* = 0.389). Intraoperative outcomes are detailed in Table 3.

### 3.4. Postoperative In-Hospital Outcomes

Overall, the postoperative course was uneventful in the great majority of patients in both age strata. No 30-day deaths, no strokes, and no new postoperative dialyses were observed in either age stratum. Given the zero event counts in modest samples, the exact (Clopper–Pearson) 95% upper confidence bound for each of these endpoints was 3.33%. The Newcombe hybrid-score 95% confidence intervals for the risk differences were +0.00% (–3.33% to +3.33%) for all three endpoints, consistent with no observable excess in either direction within the resolution of the cohort. Postoperative myocardial infarction occurred in zero elderly patients (95% CI 0.00–3.33%) and one younger patient (0.9%, 95% CI 0.02–5.01%; risk difference –0.92%, 95% CI –5.01% to +2.42%, *p* = 1.000). NOAF was infrequent and similar between groups (4.6%, 95% CI 1.51–10.38% in elderly vs. 1.8%, 95% CI 0.22–6.47% in younger; risk difference +2.75%, 95% CI –2.28% to +8.34%; *p* = 0.445; exact McNemar’s *p* = 0.45). Re-exploration for bleeding occurred in 1.8% of patients in each group (*p* = 1.000; exact McNemar’s *p* = 1.00). Postoperative coronary angiography was indicated in 21.1% of elderly and 26.6% of younger patients (*p* = 0.427); these late angiograms were symptom-driven or ischaemia-driven and were not routine surveillance procedures (typical indications were recurrent or new angina, new ischaemic changes on stress testing or imaging, and, in a small minority, investigation of unexplained heart failure or ventricular arrhythmia).

**Figure 2:**
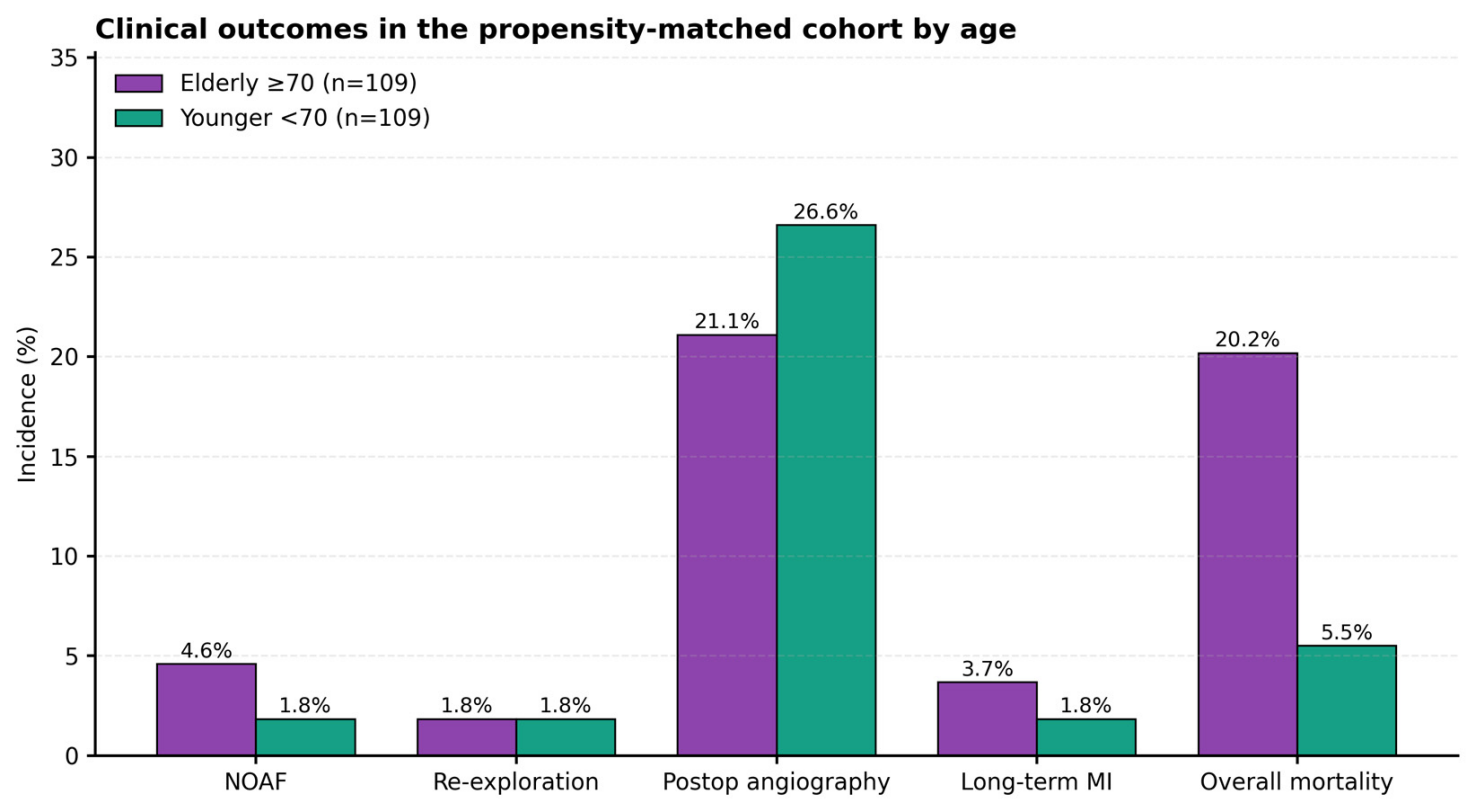
Incidence of key in-hospital and long-term clinical outcomes by age stratum in the propensity-matched cohort. Perioperative complications were equally distributed within the resolution of the cohort; the difference in overall long-term mortality is consistent with age-related life-expectancy curves over a median follow-up approaching two decades. NOAF = new-onset atrial fibrillation; MI = myocardial infarction.

Length of ICU stay was short and clinically equivalent in both groups (median 1 day [IQR 1–1] in elderly vs. 1 day [1–1] in younger, *p* = 0.146), as was the duration of mechanical ventilation (median 0 days in both groups, *p* = 0.742). Hospital length of stay was marginally longer in elderly patients (median 8 days [IQR 7–10] vs. 7 days [4,7,8,9], *p* = 0.050; Hodges–Lehmann median difference +1.0 day, bootstrap 95% CI 0.0–1.0 days). Peak postoperative CK-MB was modestly lower in elderly patients (median 30 vs. 36 U/L, *p* = 0.008); peak troponin and total CK did not differ between groups. Postoperative RBC transfusion in the ICU was similar between groups (*p* = 0.643). Postoperative outcomes are summarised in Table 4 and visualised in Figure 2.

### 3.5. Long-Term Survival and Cardiac Outcomes

**Figure 3:**
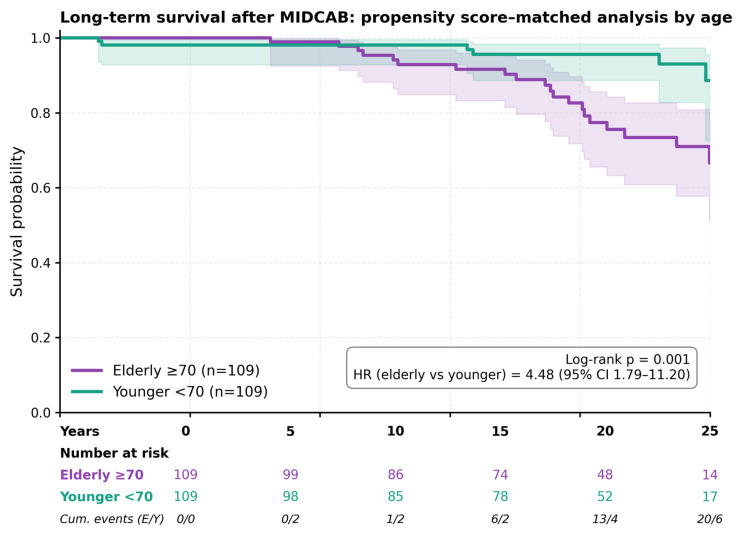
Kaplan–Meier curves for all-cause mortality stratified by age in the propensity score–matched cohort (n = 218). Shaded regions represent 95% confidence intervals. The number-at-risk table and cumulative event counts are shown below the curves. Log-rank *p* = 0.001; adjusted hazard ratio for elderly age vs. younger = 4.48 (95% CI 1.79–11.20).

Median follow-up was 19.0 years (IQR 11.5–24.3) in the elderly group and 19.6 years (12.5–24.5) in the younger group (*p* = 0.409) (Table 5); the reverse Kaplan–Meier potential median follow-up was 20.7 years and 19.9 years respectively, confirming administrative rather than premature censoring (Supplementary Figure S2). Follow-up was 100% complete for the primary endpoint of all-cause mortality. During this extended follow-up period, all-cause mortality in the matched cohort was 22 of 109 elderly patients (20.2%) versus 6 of 109 younger patients (5.5%, *p* = 0.002; exact McNemar’s *p* = 0.0025 for the matched-pair mortality endpoint; discordant pairs: 21 elderly deaths without younger-partner death; 5 younger deaths without elderly-partner death). The Kaplan–Meier estimates demonstrated a significant separation of survival curves between age groups (log-rank *p* = 0.001; Figure 3). Number-at-risk and cumulative event counts are presented within Figure 3.

Kaplan–Meier survival estimates at clinically relevant time points in the matched cohort are as follows. Elderly ≥70 years: 1-year survival 100.0%, 5-year 100.0%, 10-year 98.9% (95% CI 91.3–99.4%), 15-year 92.9% (83.2–95.9%), 20-year 82.6% (69.6–88.3%). Younger <70 years: 1-year 100.0%, 5-year 98.1% (92.7–99.5%), 10-year 98.1% (92.7–99.5%), 15-year 98.1% (92.7–99.5%), 20-year 95.6% (88.6–98.4%). These estimates are also tabulated in Supplementary Table S1.

**Figure 4:**
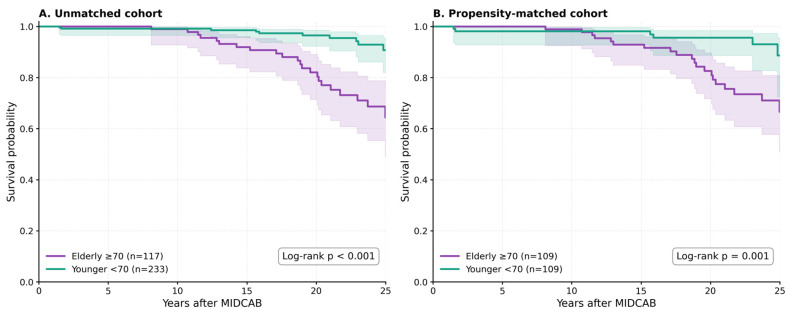
Kaplan–Meier survival curves for all-cause mortality in (**A**) the unmatched cohort (n = 350) and (**B**) the propensity score–matched cohort (n = 218). Shaded areas: 95% confidence intervals. A significant survival difference between elderly and younger patients was observed in both analyses, consistent with age-related life-expectancy curves over two decades of follow-up.

To confirm the robustness of these findings, Kaplan–Meier analysis was also performed in the full unmatched cohort, which yielded a highly significant difference between age strata (log-rank *p* < 0.001; Figure 4). Long-term myocardial infarction occurred in four elderly patients (3.7%) and two younger patients (1.8%) in the matched cohort (*p* = 0.683). Late coronary angiography was performed in only 15 patients across the entire 350-patient cohort (~4.3%) and—importantly—was undertaken on a symptom- or ischaemia-driven basis rather than as routine surveillance. In this highly selected subgroup, the LIMA-LAD anastomosis was patent and graded as good in 10 of 15 patients and occluded in 5 of 15 patients. Given the small size of the subgroup and the selection for high pre-test probability of graft or coronary pathology, these numbers should be interpreted as descriptive only and not extrapolated as a general estimate of LIMA-LAD patency in the cohort. General LIMA-LAD long-term patency expectations are based on the published literature (Loop et al. [3]; Cameron et al. [4]).

### 3.6. Multivariable Analysis of Predictors of All-Cause Mortality

**Figure 5:**
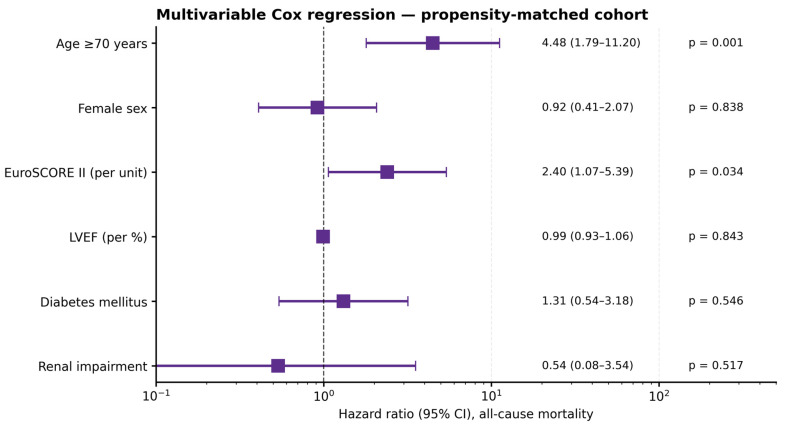
Forest plot of adjusted hazard ratios from the multivariable Cox proportional hazards regression for all-cause mortality in the propensity-matched cohort. Squares represent point estimates; horizontal lines indicate 95% confidence intervals. The dashed vertical line marks HR = 1 (no effect). Elderly age (≥70 years) and EuroSCORE II were the only variables independently associated with mortality.

To identify independent predictors of long-term all-cause mortality and to formally adjust for residual baseline differences, a multivariable Cox proportional hazards regression model was fitted in the propensity-matched cohort, including elderly age (≥70 years), female sex, EuroSCORE II, LVEF, diabetes mellitus, renal impairment, and a history of preoperative myocardial infarction. After adjustment, elderly age was strongly and independently associated with long-term mortality (adjusted HR 4.48, 95% CI 1.79–11.20, *p* = 0.001). EuroSCORE II also emerged as an independent predictor (HR 2.40 per unit, 95% CI 1.07–5.39, *p* = 0.034). Female sex (HR 0.92, *p* = 0.838), LVEF (HR 0.99 per %, *p* = 0.843), diabetes (HR 1.31, *p* = 0.546), renal impairment (HR 0.54, *p* = 0.517), and preoperative myocardial infarction history were not independently associated with the outcome (Table 6 and Figure 5).

As a pre-specified sensitivity analysis and in accordance with the events-per-variable heuristic for Cox regression, a parsimonious model restricted to three biologically motivated covariates—age ≥70 years, LVEF, and renal impairment—was fitted (events per variable ~9). To correctly reflect the matched-pair design, robust standard errors clustered on the matched-pair identifier were applied. The adjusted HR for elderly age was 4.08 (95% CI 1.65–10.08, *p* = 0.002) without clustering and 4.08 (95% CI 1.65–10.04, cluster-robust *p* = 0.002) with pair-clustering—essentially identical to the primary analysis. A univariate matched-pair Cox model with pair-cluster robust standard errors yielded HR 3.96 (95% CI 1.61–9.75, *p* = 0.003). In the sensitivity propensity model excluding EuroSCORE II (108 pairs), the parsimonious Cox HR for elderly age was 3.95 (95% CI 1.68–9.29, *p* = 0.002). Together, these analyses confirm the robustness of the association between age ≥70 years and long-term mortality across modelling choices.

To assess the robustness of these findings, an identical multivariable Cox model was fitted in the full unmatched cohort (n = 350). The results were consistent and strengthened: elderly age remained strongly associated with long-term mortality (adjusted HR 5.59, 95% CI 2.57–12.13, *p* < 0.001), with EuroSCORE II (HR 1.01 per unit, *p* = 0.039) and renal impairment (HR 2.74, *p* = 0.033) also emerging as independent predictors. This sensitivity analysis reinforces the conclusion that age ≥70 years is an independent predictor of long-term—but not perioperative—mortality after MIDCAB.

## 4. Discussion

In this propensity score–matched analysis of 350 consecutive patients undergoing MIDCAB with a median follow-up approaching two decades, we observed three principal findings. First, MIDCAB carried very low observed perioperative risk regardless of age stratum: 30-day mortality was 0% in both elderly (≥70 years) and younger patients (exact 95% upper CI 3.3% for each zero-event endpoint), and no patient developed perioperative stroke or required new postoperative dialysis. Second, perioperative and in-hospital outcomes—including operative time, transfusion requirements, NOAF, re-exploration for bleeding, ICU length of stay, and peak postoperative cardiac biomarkers—were comparable between elderly and younger patients in the matched cohort, with only a marginally longer hospital stay in the elderly group (median 8 vs. 7 days, *p* = 0.050; Hodges–Lehmann median difference +1.0 day, bootstrap 95% CI 0.0–1.0 days). Third, after multivariable adjustment, elderly age was strongly and independently associated with long-term all-cause mortality (HR 4.48; parsimonious pair-cluster model HR 4.08; sensitivity PS-without-EuroSCORE II HR 3.95—all essentially concordant), as was EuroSCORE II, while female sex, ventricular function, diabetes, and renal impairment were not. These observations are consistent with the interpretation that the long-term survival disadvantage associated with advanced age reflects age-related competing causes of death over an extended follow-up horizon rather than procedural risk attributable to MIDCAB itself. Cause-specific mortality was not directly ascertainable in the present cohort, so this interpretation is supported indirectly through comparison with published MIDCAB series in which long-term survival approximated the age-matched general population [5,6].

### 4.1. Age and Conventional CABG: A Well-Established Risk

Advancing age has long been recognised as one of the strongest predictors of adverse outcome after conventional CABG. Large registry analyses have demonstrated higher in-hospital and 30-day mortality, increased rates of stroke, renal dysfunction, and respiratory complications, as well as longer ICU and hospital stay, in elderly patients undergoing sternotomy CABG [7,8]. In a single-centre series of 2985 patients undergoing CABG that included 282 octogenarians, in-hospital mortality was 4.6% in octogenarians compared with 2.2% in septuagenarians and 2.4% in patients younger than 70 years, with respiratory failure and gastrointestinal complications occurring more frequently in the elderly [7]. Earlier series of patients aged ≥80 years undergoing conventional cardiac surgery reported in-hospital mortality rates of 6.8–8.1% after isolated CABG, two- to three-fold higher than in younger cohorts [8,14]. The GOPCABE randomised trial, comparing on- and off-pump CABG in patients aged ≥75 years, showed no benefit of either operative approach with respect to clinical outcomes at 30 days or 1 year, confirming that the substantial perioperative morbidity in this age group is largely intrinsic to sternotomy-based revascularization [9]. Age is consequently incorporated as a strong continuous risk variable in EuroSCORE II [1].

### 4.2. MIDCAB Does Not Appear to Carry the Perioperative Age Penalty of Conventional CABG

Our results suggest that the perioperative age penalty observed in conventional sternotomy CABG does not appear in the present MIDCAB cohort. This statement is made with appropriate caution given the limited number of events. In the propensity-matched cohort, 30-day mortality was 0% in both age strata (exact upper 95% CI 3.3%; Newcombe 95% CI for the risk difference –3.3% to +3.3%)—a striking finding that contrasts with the 4.6–8.1% in-hospital mortality reported in elderly conventional CABG series [7,8,14]. Equally notably, no patient developed perioperative stroke or required new postoperative renal replacement therapy within the same 95% upper confidence bound of 3.3%, regardless of age. These observations align with the 20-year Leipzig experience reported by Davierwala and colleagues, in which 2667 consecutive MIDCAB patients had an in-hospital mortality of just 0.9%, with stable outcomes despite a progressively worsening demographic risk profile over time, including increasing age and comorbidity burden [5]. Similarly, in the Australian 20-year cohort reported by Manuel and colleagues (n = 271), only one 30-day death occurred (0.4%), with isolated LAD-disease MIDCAB patients returning to an age- and sex-matched standardised mortality ratio of 0.94—that is, survival equivalent to the general population [6].

### 4.3. Why MIDCAB May Be Particularly Suited to Elderly Patients

Several physiological and technical considerations may explain why elderly patients—historically at highest risk after conventional CABG—appear to fare as well perioperatively as younger patients after MIDCAB. The avoidance of median sternotomy abolishes the risk of deep sternal wound infection and dehiscence, complications to which elderly patients with diabetes, frailty, or poor nutritional status are particularly susceptible. The avoidance of cardiopulmonary bypass eliminates the systemic inflammatory response and reduces neurocognitive injury, transfusion requirements, and renal dysfunction—all of which disproportionately affect older patients with reduced physiological reserve. The short operative time (a median of approximately 130 min in our cohort), the limited blood loss, and the rapid extubation associated with MIDCAB facilitate early mobilisation and discharge, factors that are particularly important in the elderly. The shorter hospital stay observed in our elderly group (median 8 days) is considerably better than that reported in conventional octogenarian CABG series, which typically averages 12–16 days [14]. Finally, the published literature reports high LIMA-LAD long-term patency (exceeding 90% at 10–15 years) across multiple series [3,4], independent of age, which provides an expected mechanism of durable LAD-territory protection. In the present cohort, late angiographic follow-up was available in only 15 patients (a symptom- or ischaemia-driven, and hence highly selected, subgroup) with 10 patent and 5 occluded anastomoses; this small subgroup should be interpreted as purely descriptive and not extrapolated to the whole cohort.

### 4.4. Long-Term Survival: A Reflection of Life-Expectancy Curves

As anticipated, all-cause long-term mortality was higher in elderly than in younger patients in the matched cohort (20.2% vs. 5.5%, *p* = 0.002), with an adjusted hazard ratio of 4.48 (95% CI 1.79–11.20); concordant estimates were obtained from the parsimonious pair-cluster Cox model (HR 4.08, 95% CI 1.65–10.04) and the sensitivity propensity model that excluded EuroSCORE II (HR 3.95, 95% CI 1.68–9.29). This finding is biologically inevitable: over a median follow-up of nearly 20 years, patients aged ≥70 years at the time of surgery will be aged ≥90 years at the end of follow-up, by which time age-related competing causes of death—including non-cardiac malignancy, cerebrovascular disease, dementia, and frailty-related complications—will dominate. Cause-specific mortality could not be reliably ascertained across two decades of follow-up in this single-centre cohort, and the interpretation that the elderly mortality excess is dominated by non-cardiac competing causes is therefore inferential rather than directly demonstrated. It is supported indirectly by external benchmarks: in the 20-year Australian MIDCAB series by Manuel and colleagues, MIDCAB patients with isolated LAD disease achieved a standardised mortality ratio of 0.94 against the age-matched general population [6], and in the 20-year Leipzig series, 10-year survival was 77.7%—favourable in the context of the cohort’s comorbidity burden [5]. In our own cohort, Kaplan–Meier survival at 20 years was 82.6% in the matched elderly group and 95.6% in the matched younger group (Supplementary Table S1); the elderly figure compares favourably with expected general-population survival at the same age band. In multivariable Cox regression, age was an independent predictor (HR 4.48 per ≥70 status), with EuroSCORE II also significant (HR 2.40), but neither LVEF, diabetes, renal impairment, nor sex emerged as independent predictors. This pattern is concordant with the long-term MIDCAB literature [5,6].

### 4.5. Clinical Implications

Our findings carry several practical implications. First, in elderly patients with isolated LAD disease or LAD-predominant multivessel disease in whom hybrid revascularization is planned, MIDCAB offers excellent observed perioperative outcomes in appropriately selected patients treated at an experienced centre, with no 30-day deaths in either age stratum (upper 95% CI 3.3%) and no evidence of an excess in complications compared with younger patients within the resolution of the cohort. Age alone should not be a contraindication to MIDCAB; rather, a comprehensive assessment of frailty and comorbidity—informed by EuroSCORE II [1] and contemporary geriatric evaluation tools—should guide patient selection. Second, although EuroSCORE II appropriately incorporates age as a continuous risk variable [1], the predicted operative mortality may overestimate actual MIDCAB-specific perioperative risk in elderly patients; risk-prediction tools may need to be recalibrated for minimally invasive coronary surgery in this population, as has previously been suggested in the elderly by Faerber and colleagues for off-pump CABG [10]. Third, in the era of expanding hybrid revascularization strategies and an ageing population, MIDCAB represents a particularly attractive option for older patients who might otherwise be considered prohibitively high risk for sternotomy CABG and in whom percutaneous-only strategies provide inferior long-term LAD-territory protection. This recommendation is consistent with current 2018 ESC/EACTS guidelines on myocardial revascularization [12].

### 4.6. Limitations

Several limitations of this study should be acknowledged. First, the analysis is retrospective and single-centre; despite the use of propensity score matching, residual confounding from unmeasured variables (for example, frailty indices, sarcopenia, cognitive function, nutritional status, socioeconomic factors, and individual patient preferences) cannot be excluded. Frailty and cognitive assessments in particular were not systematically recorded across the entire study period, and the resulting absence of frailty-adjusted analysis is a genuine limitation given the recognised primacy of frailty as a predictor of cardiac-surgical outcome in elderly patients. Body mass index, preoperative anaemia, mobility, chronic obstructive pulmonary disease severity, and LAD target-vessel calibre and calcification were similarly not systematically captured for the whole cohort and are acknowledged as unmeasured confounders. Second, the relatively modest number of elderly patients (n = 117) limits the statistical power to detect rare perioperative events; nevertheless, the consistent finding of 0% 30-day mortality across two decades is striking, with an exact upper 95% CI of 3.3% for each zero-event perioperative endpoint that quantifies the residual uncertainty. Third, the matched analysis required exclusion of 8 unmatched elderly patients (93.2% matching rate); this minor loss of data is unlikely to have biased the findings. Fourth, the chosen age cutoff of 70 years was selected a priori but is somewhat arbitrary; alternative cutoffs (65 or 75 years) or treating age as a continuous variable might have yielded different effect sizes, although the underlying age–mortality relationship is monotonic and would be unlikely to alter the qualitative conclusions. Fifth, late coronary angiography was performed in a minority of patients (n = 15 in the entire cohort, on a symptom- or ischaemia-driven rather than routine basis), precluding firm conclusions about age-specific LIMA-LAD patency. Sixth, cause-specific mortality (cardiac vs. non-cardiac) could not be reliably ascertained for all patients given the very long follow-up—a particularly relevant limitation in the elderly group, where non-cardiac competing causes of death predominate over two decades. Seventh, selection bias arising from Heart Team preferences during the study period is a genuine and probably substantial contributor to the low observed elderly perioperative mortality; specifically, elderly patients with prohibitive systemic risk profiles were typically redirected to percutaneous coronary intervention or medical therapy, so the elderly MIDCAB cohort is enriched for a comparatively favourable non-cardiac risk profile. Eighth, the mixed cohort of isolated LAD disease (64.9%) and LAD-predominant multivessel disease (35.1%)—a subset of whom underwent hybrid revascularization with staged non-LAD percutaneous coronary intervention—introduces heterogeneity in completeness of revascularization that we could not fully adjudicate; the completeness-of-revascularization data (whether residual non-LAD disease was managed medically or by staged PCI) were not captured as a discrete field in our database. Finally, all procedures were performed at a single tertiary centre by an experienced minimally invasive cardiac surgical team, and generalisability to lower-volume centres should be made with caution.

## 5. Conclusions

In this propensity score–matched analysis of patients undergoing MIDCAB with up to two decades of follow-up, elderly age (≥70 years) was not associated with adverse perioperative outcome. Thirty-day mortality was 0% in both age strata (exact upper 95% CI 3.3%), major perioperative complications were rare and equally distributed within the resolution of the cohort, and intraoperative and ICU outcomes were comparable. The long-term mortality excess in the elderly (adjusted HR 4.48; concordant with parsimonious HR 4.08 and sensitivity-PS HR 3.95) is consistent with age-related life-expectancy curves in the source population; cause-specific mortality was not directly ascertainable in the present cohort. These results support MIDCAB as a feasible revascularization strategy associated with favourable observed early outcomes and long-term survival consistent with the published MIDCAB literature, in appropriately selected elderly patients with single-vessel and LAD-predominant coronary artery disease treated at experienced centres, and argue against using chronological age alone as a basis for declining surgical revascularization in appropriately selected older patients.

## Figures and Tables

**Table 1:** Baseline characteristics of the entire MIDCAB cohort before propensity score matching (n = 350).

Variable	Elderly ≥70 (n = 117)	Younger <70 (n = 233)	*p*-Value	SMD
Age, years	75.4 ± 4.1	57.6 ± 8.3	<0.001	2.497
Female sex	43 (36.8%)	59 (25.3%)	0.036	0.249
EuroSCORE II	0.9 (0.8–1.2)	0.8 (0.7–1.1)	<0.001	−0.066
LVEF, %	56.4 ± 5.3	57.9 ± 5.6	0.018	−0.266
Urgency status, n (%)				
Elective	95 (81.2%)	181 (77.7%)		
Urgent	18 (15.4%)	43 (18.5%)		
Emergency	4 (3.4%)	9 (3.9%)		
(overall urgency *p*-value)			0.747	
Single-vessel disease (LAD)	75 (64.1%)	152 (65.2%)	0.928	−0.024
Two-vessel disease	28 (23.9%)	51 (21.9%)	0.767	0.049
Three-vessel disease	14 (12.0%)	30 (12.9%)	0.943	−0.028
Recent MI (<90 days)	5 (4.3%)	16 (6.9%)	0.468	−0.113
STEMI	0 (0.0%)	4 (1.7%)	0.305	−0.187
NSTEMI	5 (4.3%)	15 (6.4%)	0.563	−0.096
Prior PCI	22 (18.8%)	64 (27.5%)	0.100	−0.207
Smoking	28 (23.9%)	77 (33.0%)	0.103	−0.203
Diabetes (any)	25 (21.4%)	36 (15.5%)	0.220	0.153
Non-insulin-dependent DM	21 (17.9%)	31 (13.3%)	0.321	0.128
Insulin-dependent DM	4 (3.4%)	5 (2.1%)	0.489	0.077
Arterial hypertension	105 (89.7%)	211 (90.6%)	0.959	−0.027
Hyperlipidemia	60 (51.3%)	120 (51.5%)	1.000	−0.004
Renal impairment	9 (7.7%)	18 (7.7%)	1.000	−0.001
Preoperative dialysis	0 (0.0%)	3 (1.3%)	0.554	−0.162

Values are mean ± SD, median (IQR) or n (%). *p*-values from Welch’s *t*-test, Mann–Whitney U, χ^2^ or Fisher’s exact test as appropriate. SMD = standardized mean difference; |SMD| < 0.10 indicates negligible imbalance.

**Table 2:** Baseline characteristics of the propensity score–matched cohort (n = 218; 109 pairs).

Variable	Elderly ≥70 (n = 109)	Younger <70 (n = 109)	*p*-Value	SMD
Age, years	75.4 ± 4.1	58.2 ± 8.2	<0.001	2.663
Female sex	38 (34.9%)	40 (36.7%)	0.888	−0.038
EuroSCORE II	0.9 (0.7–1.2)	0.9 (0.7–1.1)	0.136	0.011
LVEF, %	56.7 ± 5.3	57.3 ± 5.5	0.376	−0.120
Urgency status, n (%)				
Elective	89 (81.7%)	88 (80.7%)		
Urgent	17 (15.6%)	17 (15.6%)		
Emergency	3 (2.8%)	4 (3.7%)		
(overall urgency *p*-value)			0.928	
Single-vessel disease (LAD)	72 (66.1%)	64 (58.7%)	0.328	0.152
Two-vessel disease	23 (21.1%)	26 (23.9%)	0.746	−0.066
Three-vessel disease	14 (12.8%)	19 (17.4%)	0.450	−0.128
Recent MI (<90 days)	4 (3.7%)	3 (2.8%)	1.000	0.052
STEMI	0 (0.0%)	0 (0.0%)	1.000	0.000
NSTEMI	4 (3.7%)	3 (2.8%)	1.000	0.052
Prior PCI	21 (19.3%)	19 (17.4%)	0.861	0.047
Smoking	28 (25.7%)	26 (23.9%)	0.875	0.043
Diabetes (any)	19 (17.4%)	18 (16.5%)	1.000	0.024
Non-insulin-dependent DM	15 (13.8%)	14 (12.8%)	1.000	0.027
Insulin-dependent DM	4 (3.7%)	4 (3.7%)	1.000	0.000
Arterial hypertension	97 (89.0%)	100 (91.7%)	0.646	−0.093
Hyperlipidemia	57 (52.3%)	53 (48.6%)	0.684	0.073
Renal impairment	7 (6.4%)	11 (10.1%)	0.460	−0.134
Preoperative dialysis	0 (0.0%)	0 (0.0%)	1.000	0.000

After matching, all standardized mean differences for covariates included in the propensity model were below 0.20, indicating adequate balance. The large persistent SMD for age is expected, as age was the exposure variable and was not included in the propensity model. Statistical conventions as in Table 1.

**Table 3:** Intraoperative outcomes in the propensity score–matched cohort.

Variable	Elderly ≥70 (n = 109)	Younger <70 (n = 109)	*p*-Value
Operative time, min	130.1 ± 35.1	122.5 ± 30.9	0.091
Intraop RBC transfusion, units	0.0 (0.0–0.0)	0.0 (0.0–1.0)	0.102
Intraop platelet transfusion, units	0.0 (0.0–0.0)	0.0 (0.0–0.0)	0.389
Intraop RBC transfusion (≥1 unit), n (%)	22 (20.2%)	32 (29.4%)	0.158

Continuous variables are mean ± SD or median (IQR); categorical variables are n (%). RBC = red blood cell.

**Table 4:** Postoperative in-hospital outcomes in the propensity score–matched cohort.

Variable	Elderly ≥70 (n = 109)	Younger <70 (n = 109)	*p*-Value
ICU length of stay, days	1.0 (1.0–1.0)	1.0 (1.0–1.0)	0.146
Mechanical ventilation, days	0.0 (0.0–0.0)	0.0 (0.0–0.0)	0.742
Hospital length of stay, days	8.0 (7.0–10.0)	7.0 (6.0–9.0)	0.050
Max postoperative CK-MB, U/L	30.0 (24.0–41.0)	36.0 (26.0–43.0)	0.008
Max postoperative CK, U/L	425.0 (347.0–626.0)	426.0 (350.0–626.0)	0.598
Max postoperative troponin	28.0 (21.0–37.0)	28.0 (20.0–34.0)	0.496
ICU RBC transfusion, units	0.0 (0.0–0.0)	0.0 (0.0–0.0)	0.643
New-onset atrial fibrillation	5 (4.6%)	2 (1.8%)	0.445
Re-exploration for bleeding	2 (1.8%)	2 (1.8%)	1.000
New postoperative dialysis	0 (0.0%)	0 (0.0%)	1.000
Postoperative stroke	0 (0.0%)	0 (0.0%)	1.000
Postoperative MI	0 (0.0%)	1 (0.9%)	1.000
Postop angiography required	23 (21.1%)	29 (26.6%)	0.427

Values are median (IQR) for continuous variables and n (%) for categorical variables. For rare or zero-event endpoints, exact (Clopper–Pearson) 95% CIs and Newcombe 95% CIs for the between-group risk difference are given in the accompanying text (Section 3.4). CK = creatine kinase; ICU = intensive care unit; MI = myocardial infarction; NOAF = new-onset atrial fibrillation; RBC = red blood cell.

**Table 5:** Long-term follow-up outcomes in the propensity score–matched cohort.

Variable	Elderly ≥70 (n = 109)	Younger <70 (n = 109)	*p*-Value
Follow-up, years	19.0 (11.5–24.3)	19.6 (12.5–24.5)	0.409
30-day mortality	0 (0.0%)	0 (0.0%)	1.000
All-cause mortality (overall)	22 (20.2%)	6 (5.5%)	0.002
Long-term MI	4 (3.7%)	2 (1.8%)	0.683

Thirty-day mortality is defined as death within 30 days of surgery. Median follow-up exceeded 19 years in both groups; the reverse Kaplan–Meier potential median follow-up was 20.7 and 19.9 years in the elderly and younger groups respectively. Kaplan–Meier survival estimates at 1, 5, 10, 15, and 20 years are provided in Supplementary Table S1.

**Table 6:** Multivariable Cox proportional hazards regression for all-cause mortality in the propensity-matched cohort.

Variable	Adjusted HR	95% CI	*p*-Value
Age ≥70 years	4.48	1.79–11.20	0.001
Female sex	0.92	0.41–2.07	0.838
EuroSCORE II (per unit)	2.40	1.07–5.39	0.034
LVEF (per %)	0.99	0.93–1.06	0.843
Diabetes mellitus	1.31	0.54–3.18	0.546
Renal impairment	0.54	0.08–3.54	0.517

Outcome: all-cause mortality. Preoperative MI history is omitted from the forest plot because no events occurred in patients with this exposure. The EuroSCORE II per-unit HR (2.40) is scaled to a 1-unit absolute change; in the matched cohort the observed EuroSCORE II range is 0.52–3.61 with SD 0.57 units, so a 1-unit change represents ~1.75 SDs and hence a large HR. In the unmatched cohort, one clear outlier (a value of 175, likely a data-entry artefact) inflates the SD to 9.31 units, so the per-unit HR of 1.01 reflects the small fraction of the observed range that a 1-unit change represents. HR = hazard ratio; CI = confidence interval; LVEF = left ventricular ejection fraction; MI = myocardial infarction.

## Data Availability

The data presented in this study are available on reasonable request from the corresponding author. The data are not publicly available owing to privacy and ethical restrictions.

## Supplementary Materials

The following supporting information can be downloaded at: https://www.mdpi.com/article/10.3390/jcm15156043/s1, Figure S1: Patient flow diagram, Figure S2: Reverse Kaplan–Meier estimate of potential median follow-up, Table S1: Kaplan–Meier survival estimates at 1, 5, 10, 15, and 20 years with 95% confidence intervals in the propensity score–matched cohort.
