# Supplementary material for: Early and Long-Term Outcomes of Minimally Invasive Direct Coronary Artery Bypass in Elderly Patients: A Propensity Score-Matched Analysis
Source: J Clin Med. 2026 Aug 3;15(15):6043. doi: 10.3390/jcm15156043 (PMC13466365; doi:10.3390/jcm15156043)
Supplement: Supplementary file 1 [file jcm-15-06043-s001.zip › jcm-4392932-supplementary.pdf]

Supplementary Materials

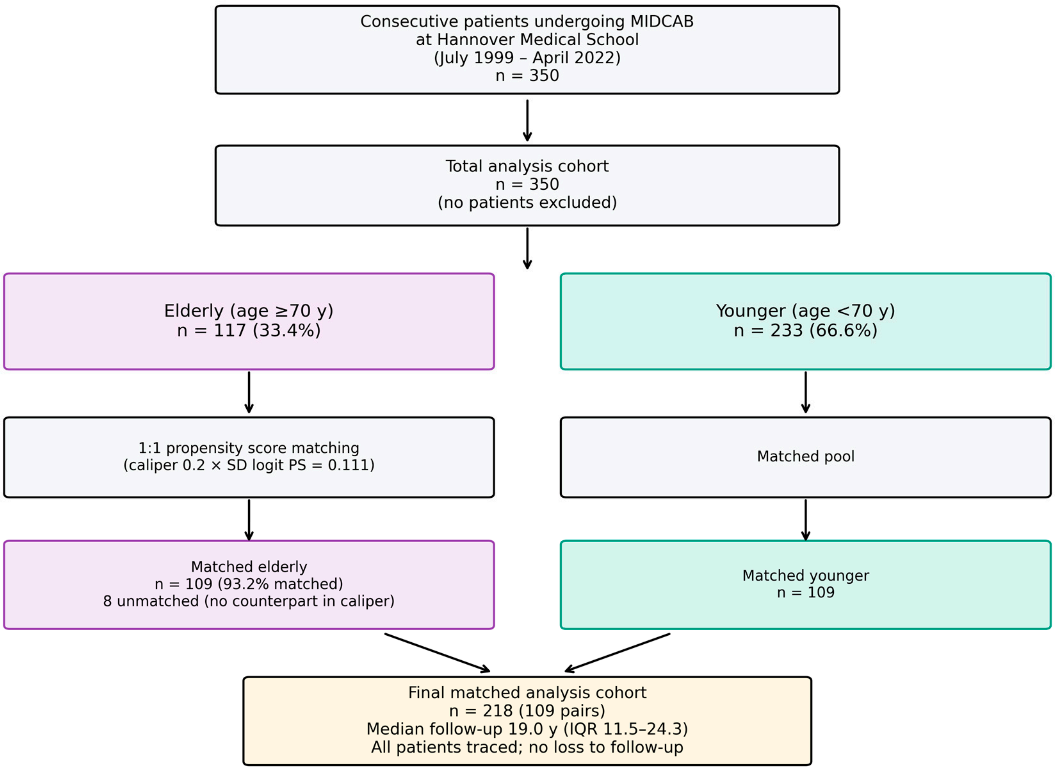

Supplementary Figure S1. Patient flow diagram showing consecutive MIDCAB patients at Hannover Medical School (July 1999 – April 2025), age stratification, 1:1 propensity score matching with caliper 0.2 × SD of the logit propensity score (= 0.111), and the final matched analysis cohort. Follow-up completeness was 100%.

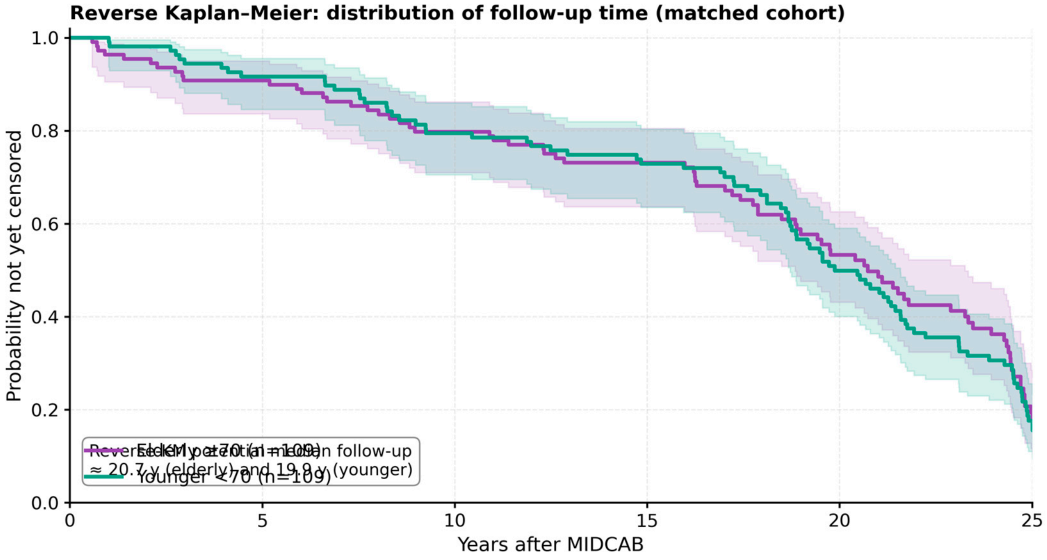

Supplementary Figure S2. Reverse Kaplan–Meier estimate of the censoring distribution (matched cohort). The potential median follow-up was 20.7 years in the elderly group and 19.9 years in the younger group, confirming

administrative censoring at study end rather than premature loss to follow-up.

Supplementary Table S1. Kaplan–Meier survival estimates with 95% confidence intervals at clinically relevant time points (matched cohort).

Time point	Elderly ≥70 (survival)	95% CI	Younger <70 (survival)	95% CI
1 year	100.0%	(–)	100.0%	(–)
5 years	100.0%	(–)	98.1%	(92.7–99.5%)
10 years	98.9%	(91.3–99.4%)	98.1%	(92.7–99.5%)
15 years	92.9%	(83.2–95.9%)	98.1%	(92.7–99.5%)
20 years	82.6%	(69.6–88.3%)	95.6%	(88.6–98.4%)

Survival estimates from the Kaplan–Meier method with 95% confidence intervals computed by the exponential Greenwood formula. Elderly group: n = 109, 22 deaths; younger group: n = 109, 6 deaths.
